# Violence against women and girls: more RESPECT needed

**DOI:** 10.1016/j.lana.2024.100696

**Published:** 2024-02-13

**Authors:** The Lancet Regional Health – Americas

Every 11 minutes, somewhere in the world, another woman or girl is murdered by a family member or intimate partner. No matter how the numbers are expressed, the statistics are shocking: nearly 50,000 women or girls are murdered annually by someone in their family circle. While the number of murders decreased by 13% from 2010 to 2020 in Europe, it increased by 9% in the Americas over the same period. As if this were not enough, it is estimated that almost a third of women aged 15 years or older worldwide suffered physical and/or sexual violence in their lifetime, which amounts to more than 700 million women. Most sexual assaults seem to occur within romantic or sexual relationships.

Gender-based violence includes psychological, physical, and sexual violence. The latter includes any of its forms and child sexual abuse. As with many other public health issues, violence against women and girls is out of control in all countries and some are affected more than others. For women, Honduras, Dominican Republic, El Salvador, Bolivia, and Brazil are the most unsafe countries in Latin America, along with Belize and Guyana in the Caribbean, according to UN estimates. To put it into perspective, the proportion of femicide in Honduras is 4.6 cases per 100,000 women, compared with 0.66 in Sweden, one of the safest countries in the world for women.

Little is known about the trends in the prevalence of physical or sexual violence in the Americas or globally. A study using data from 53 low-income and middle-income countries between 2000 and 2021 showed that the prevalence of any type of intimate partner violence affecting women aged 15–49 years old in the past 12 months was 37%. This prevalence is nearly three times the estimated global prevalence in the same age group (13%). There was a high variability in the prevalence of physical, sexual, and psychological violence across countries and regions. Data from the five included countries of the Americas revealed that physical violence was more prevalent than sexual or psychological violence. However, there was a consistent increase in psychological intimate partner violence in most regions. A small study in Panama showed that among adolescents, psychological violence was far more prevalent than sexual or physical violence. Overall, the accumulated evidence to date reminds us of the need for further and more granular research for the region.

Gender-based violence is linked to hostile and unsafe environments, but it is also linked to an unfortunately persistent culture of the dominant position of men that still occurs at different levels: at home, at work, within governments, at sports. The belief in a hegemonic masculinity is still profound among men and boys and even accepted by women and men as the norm. Despite all the progress in technology and information access, our societies are still far behind from being a civilised world that is respectful of women and girls. It has been 30 years since the UN Declaration on the Elimination of Violence against Women, but the statistics remain alarming. In response to the growing problem, WHO and UN Women proposed the RESPECT framework in 2019 to tackle the root causes of violence against women and girls. This framework proposes seven targets: Relationship skills strengthened; Empowerment of women; Services ensured to survivors; Poverty reduced; Environments made safe; Child and adolescent abuse prevented; and Transformed attitudes, beliefs, and norms. Naturally, given the increase in the number of murders and gender-based psychological violence in many countries, a key question is whether the existing programs and several proposed frameworks have been fully implemented or are actually effective in the regions that have not seen an improvement.

Less than 80% of the countries in the world have passed legislation to address domestic violence. In addition, it is believed that only 40% of women seek help after a traumatic event and only 10% report it to police. Helplines have been implemented by countries to assist victims. However, experts and governments cannot directly help a victim if they have not been contacted. In 1993, Peru began the construction of Women's Emergency Centres (CEM) to offer psychological, social, and legal support to victims of violence. To date, there are more than 400 centres nationwide. CEMs assisted nearly 30 000 women between January and April, 2023. More efforts are needed in our region, and globally, to build trust between women experiencing violence and authorities, to supress stigma, to enforce laws, to banish impunity, to better impart values to children and adolescents, and to create support centres.

Programs and comprehensive frameworks—like RESPECT—are paramount to tackle gender-based violence and prevent murders. Policymakers, in coordination with public health experts, should ensure that programs are implemented, monitored, and evaluated. More data are needed to better understand the magnitude of this crisis, the context of the violence, the characteristics of the perpetrators, and other triggering factors. Better support for victims could also facilitate their determination to speak out and for the authorities to understand the real dimension of the probably underestimated numbers. Lawmakers and law enforcement also have a fundamental role, as offenders and murderers are usually re-incidents. Although RESPECT and other similar frameworks have been published, the reduction and elimination of gender-based violence will not be achieved if programs are not implemented or feasible to implement for a given population. RESPECT is expected to be adopted by countries soon. However, no implemented program can be assumed to be effective if it is not monitored and evaluated. There is still much hard work to be done.

